# Construction of Pickering Emulsion of *Amomum tsaoko* Essential Oil Based on Cellulose Nanocrystals to Enhance Pullulan Film: Structure, Antibacterial Property, and Preservation Effect of Mango

**DOI:** 10.3390/foods15132282

**Published:** 2026-06-25

**Authors:** Lin Zhu, Jiameng Liu, Zhikai Zhuang, Shaokai Zhang, Lijing Lin

**Affiliations:** Hainan Key Laboratory of Storage and Processing of Fruits and Vegetables, Agricultural Products Processing Research Institute, Chinese Academy of Tropical Agricultural Sciences, Zhanjiang 524001, China; zl13994152906@126.com (L.Z.); jmengliu@163.com (J.L.); zhuangkai_612@163.com (Z.Z.); shaokai_zsk@163.com (S.Z.)

**Keywords:** cellulose nanocrystals, Pullulan, Pickering emulsion of *Amomum tsaoko* essential oil, preservation packaging

## Abstract

Mango is prone to postharvest microbial infection, which leads to deterioration. Although essential oils show great potential for antibacterial and antioxidant applications, their effectiveness is limited by poor stability. In this study, cellulose nanocrystal (CNC)-stabilized *Amomum tsaoko* essential oil emulsion (AEO–CNC) was incorporated into a pullulan (Pul) matrix through hydrogen bonding to prepare antibacterial films. A stable AEO–CNC emulsion was obtained by precisely adjusting the AEO concentration. The effects of emulsion incorporation on microstructure evolution, molecular interactions, physiochemical properties, and biological functionalities of Pul-based composite films were systematically investigated, and their preservation effect on mango was evaluated. With the increasing incorporation of AEO Pickering emulsion, the ultraviolet-blocking property (transmittance <30%), antioxidant capacity (46.83%), and antibacterial activity of PP-CNC-AEO films were significantly improved. In addition, these films exhibited excellent mechanical properties (tensile strength of 30.62 MPa) and thermal stability. The PP-CNC-AEO films had a more uniform and compact surface structure, with no bubbles and fewer internal pores, which indicates the formation of an effective cross-linked network. When PP-CNC-50%AEO-F was used to preserve mango, it reduced weight loss, delayed the increase in soluble solids, and postponed the post-ripening process, exhibiting strong potential for extending the shelf life of fruit.

## 1. Introduction

At present, most packaging films available on the market are made from petroleum-based materials, which are non-renewable and difficult to degrade. These materials may contain combustion additives, heavy metals, and oils. Small molecular oligomers can easily migrate into food and may produce harmful substances when incinerated in large quantities. Therefore, the development of safe, biodegradable, and renewable packaging materials is imperative. Biopolymer films are among the most promising candidates for sustainable packaging. Derived from protein, polysaccharides, or lipids, these films are biodegradable and can be loaded with bioactive substances with antioxidant and antibacterial properties [1]. Mango is an important tropical economic fruit rich in many nutrients and secondary metabolites such as vitamins and phenolic compounds. However, it is a typical climacteric fruit, and ethylene production accelerates postharvest ripening and senescence. In addition, mango is easily infected by pathogenic fungi during postharvest storage and transportation [2]; its high susceptibility to rot makes preservation a major challenge. Therefore, recent research has gradually focused on combining natural antibacterial agents and antioxidant compounds, with packaging technologies to delay postharvest quality deterioration and extend mango shelf life.

*Amomum tsaoko*, a perennial herb belonging to the Zingiberaceae family, contains various bioactive phytochemicals. It is traditionally used to treat nausea, vomiting, and dyspepsia [3]. It also has potential medical value and adds a certain flavor to food [4]. *A. tsaoko* essential oil (AEO) is a volatile aromatic compound extracted from the plant and has antibacterial [5], antioxidant [6], hypoglycemic, anti-inflammatory [3], anti-infective [7], and antitumor properties [8]. AEO is mainly composed of monoterpenoids and oxidized monoterpenoids, such as pinene, linalool, 1,8- cineole, terpineol, citronellol, and citronellal, with 1,8- cineole being the most important component [9,10]. As a natural source of bioactive substances, essential oils (EOs) are widely used as antibacterial and antioxidant agents to enhance the performance of packaging films. However, EOs are prone to oxidation when exposed to the environment, making it necessary to choose suitable carriers and improve their stability in food packaging. Currently, most studies preparing EO-loaded films use surfactant compounds to facilitate mixing of EO and the film-forming system, but such compounds may have potential adverse effects on the environment and human health. Alternatively, directly incorporating the EO into the film-forming solution often leads to issues such as degradation, volatilization, and solvent phase separation because of incompatibility between the hydrophobic oil and hydrophilic substrate [11].

Pickering emulsions, as a stable nanoparticle-based delivery system derived from natural biopolymers, exhibit good compatibility with bio-based film-forming substrates. The formation of Pickering emulsions does not require the addition of any chemical stabilizer, making them non-toxic and highly promising for food preservation. Additionally, reports have proved that using Pickering emulsion as a carrier to incorporate Eos into films is beneficial for improving film performance. For example, Pickering emulsions containing *Zanthoxylum bungeanum* EO exhibit improved oxidation resistance and compatibility [12]. Similarly, cross-linked gelatin films prepared through the solution casting method, incorporating zein/pectin-embedded Pickering emulsions of cinnamon EO, have excellent mechanical properties [13]. Despite recent advances, many existing film systems rely on the incorporation of diverse components, such as modified starch [11], proteins [14] and modified proteins [15], into heterogeneous polymer matrices. This compositional disparity often leads to poor interfacial compatibility, compromising the structural integrity and mechanical performance of the resulting composite films. Moreover, although conventional EOs such as ginger [16] and cinnamon [15] have been extensively investigated for food preservation applications, the use of Pickering emulsions for encapsulating AEO and enhancing the postharvest preservation of tropical fruits remains relatively limited. EO emulsions allow controlled release through nanocarriers or specific composite film matrices, thus prolonging antibacterial action. AEO is incorporated into polysaccharide-based films through Pickering emulsion technology, which is expected to mitigate its volatility and facilitate sustained release. Consequently, the enhanced synergistic antioxidant and antibacterial effects are expected to improve fruit preservation efficacy and significantly prolong the shelf life of mangoes. However, the effects of different proportions of AEO Pickering emulsions on the physicochemical, mechanical, and functional characteristics of Pullulan (Pul) films remain unclear.

Active packaging films mainly function by releasing active substances in the film into the headspace of food or its packaging environment. However, the instability of active substances limits their antibacterial and antioxidant effectiveness. Therefore, a Pickering emulsion stabilized with cellulose nanocrystals (CNCs) was prepared to encapsulate AEO and incorporated into a Pul substrate to prepare composite films. In contrast to conventional heterogeneous systems, CNC and Pul are naturally derived polysaccharides enriched with hydroxyl groups. This structural similarity promotes the formation of a robust, highly interconnected hydrogen-bonding network, effectively mitigating interfacial defects and improving film cohesion. Consequently, the resulting matrix enables highly efficient and controlled release of AEO. The optimal concentration of AEO Pickering emulsion was determined in preliminary experiments, and its stability was assessed. The mechanical properties, hydrophilicity, ultraviolet (UV)-blocking ability, thermal stability, oxidation resistance, and in vitro antibacterial activity of the film were also comprehensively evaluated. Scanning electron microscopy (SEM), Fourier transform infrared spectroscopy (FT-IR), and X-ray diffraction (XRD) were used to study the effects of emulsion incorporation on the microstructure, intermolecular interaction, and crystalline structure of the films. Moreover, considering mango as a model fruit, key quality parameters during storage, including appearance, weight loss, and total soluble solids (TSS), were monitored to assess the impact on fruit quality. This study provides fundamental data and theoretical support for improving postharvest storage and transportation of fruits and vegetables.

## 2. Materials and Methods

### 2.1. Materials and Reagents

AEO and PBS buffer were purchased from Shanghai Yuanye Biotechnology Co., Ltd. (Shanghai, China); CNCs (Length 200 nm, diameter 10 nm) were purchased from Shansi Technology (Guangzhou, China); Pullulan (Pul) and sodium hydroxide (NaOH) were purchased from Shanghai Aladdin Biochemical Technology Co., Ltd. (Shanghai, China); glycerol was purchased from Annaiji Chemical (Shanghai, China); calcium chloride was purchased from Guangdong Guanghua Technology Co., Ltd. (Shantou, China); Nile red and dimethyl sulfoxide were purchased from Shanghai McLean Biochemical Technology Co., Ltd. (Shanghai, China); hydrochloric acid (HCL) was purchased from Guangdong Guangshiji Technology Co., Ltd. (Guangzhou, China); anhydrous ethanol and methanol were purchased from Xilong Science Co., Ltd.(Guangzhou, China).; LB broth, LB agar, *Escherichia coli* CMCC(B)44102 and *Staphylococcus aureus* CMCC(B)26003 were all purchased from Guangdong Huankai Microbial Technology Co., Ltd. (Guangzhou, China); the Macmillan turbidimetric tube was purchased from Changde Beekman Biotechnology Co., Ltd. (Changde, China); the DPPH detection kit was purchased from Suzhou Grace Biotechnology Co., Ltd. (Suzhou, China); the ABTS test kit was purchased from Shanghai Yuanye Biotechnology Co., Ltd. (Shanghai, China); and fresh mangoes were purchased from Jiangnan Fruit and Vegetable Wholesale Market in Zhanjiang City.

### 2.2. Preparation of Pickering Emulsion of AEO

A 1 wt% CNC suspension was prepared and ultrasonically dispersed for 20 min using a digital ultrasonic cleaner (Dongguan Fushijie Ultrasonic Technology Co., Ltd. PL-S40, China) at a frequency of 40 kHz and an estimated output power of 240 W to form the aqueous phase. Subsequently, 1–5 wt% AEO was added to the CNC suspension, followed by homogenization using a hand-held homogenizer for 3 min (Hangzhou Miou Instrument Co., Ltd. MT-30K, Hangzhou, China) at room temperature at 22,000 rpm. The crude emulsion was homogenized by a micro-jet high-pressure homogenizer (AST/AMF-5 of Antuosi Nanotechnology (Suzhou) Co., Ltd., Suzhou, China), and after six cycles at 800 bar, the Pickering emulsion of AEO was finally obtained.

### 2.3. Droplet Size, PDI, and Zeta Potential of AEO Pickering Emulsion

The droplet size, polydispersity index (PDI), and zeta potential of the nanoemulsion were measured in triplicate at 25 °C using a Zetasizer Nano ZSP (Malvern Panalytical, Worcestershire, UK) [17]. All experiments were conducted in triplicate.

#### Study on Microstructure and Stability of AEO Pickering Emulsion

A 0.1% (*w*/*v*) Nile red staining solution was prepared in dimethyl sulfoxide (DMSO). Subsequently, 20 μL of the Nile red solution was added to 0.5 mL of a 2 wt% AEO Pickering emulsion (AEO–CNC) and incubated in the dark for 20 min to prevent photobleaching. The stained emulsion was then mounted on a glass slide, covered with a coverslip, and the oil phase distribution was observed using a laser scanning confocal microscope (LSM 800, Zeiss, Oberkochen, Germany) with an excitation wavelength of 488 nm [18].

For heat treatment, a 2 wt% AEO Pickering emulsion (AEO–CNC) was placed in test tubes and incubated for 30 min in a constant temperature water bath (HH-6, Shanghai Lichen Instrument Technology Co., Ltd., Shanghai, China) at 20, 40, 60, 80, and 100°C, respectively. For pH treatment, the pH of the AEO–CNC emulsion was adjusted to 2, 4, 6, 8, and 10 with NaOH or HCl solution. The droplet size (Z-mean) and zeta potential of the nanoemulsion were then measured using a nanoparticle size analyzer, with all measurements performed in triplicate.

### 2.4. Preparation and Characterization of Films

Pullulan (Pul, powder form) (4 g) was dissolved in 100 mL deionized water and stirred using a magnetic stirrer (TP-350E+; Hangzhou Miou Instrument Technology Co., Ltd., Hangzhou, China) for 2 h at room temperature to obtain a 4 wt% Pul solution. Glycerol (0.25 wt%) was added as a plasticizer. Subsequently, an appropriate amount of AEO–CNC was incorporated to achieve final concentrations of 0, 35, 40, 45, and 50 wt% in the film-forming solutions. The resulting films were designated as PP-F, PP-CNC-35% AEO-F, PP-CNC-40% AEO-F, PP-CNC-45% AEO-F, and PP-CNC-50%AEO-F, respectively.

Simultaneously, a 2 wt% AEO solution was directly added to the Pul solution to prepare control films. The final AEO concentrations in the film-forming solutions were adjusted to 35, 40, 45, and 50 wt%, and the resulting films were designated as PP-35% AEO-F, PP-40% AEO-F, PP-45% AEO-F, and PP-50%AEO-F, respectively. The solution was dried at 40 °C for 48 h, and the resulting film was stored at 25°C under 50% relative humidity for subsequent analyses. See Table 1 for sample thickness.

**Table 1:** Thin film thickness table. ^a–d^ refers to the significant differences between different groups (*p* < 0.05).

Films	Thickness
PP-F	0.128 ± 0.007 ^abc^
PP-CNC-35%AEO-F	0.108 ± 0.019 ^cd^
PP-CNC-40%AEO-F	0.120 ± 0.013 ^bcd^
PP-CNC-45%AEO-F	0.104 ± 0.016 ^d^
PP-CNC-50%AEO-F	0.104 ± 0.016 ^d^
PP-35%AEO-F	0.124 ± 0.014 ^abcd^
PP-40%AEO-F	0.124 ± 0.010 ^abcd^
PP-45%AEO-F	0.136 ± 0.010 ^ab^
PP-50%AEO-F	0.146 ± 0.010 ^a^

#### 2.4.1. Mechanical Properties of the Film

PP-F and PP-CNC-(35–50%) AEO-F films were cut into rectangular strips (1.0 cm × 4.0 cm). The tensile strength (TS) and elongation at break (EAB) were measured using a texture analyzer (Stable Micro Systems TA.XT Plus, Surrey, UK) with a contact force of 10 g and a test speed of 1 mm/s [19]. All experiments were conducted in quintuplicate.

#### 2.4.2. Moisture Content (MC) of the Film

PP-F and PP-CNC-(35–50%) AEO-F films were cut into 5.0 cm × 5.0 cm samples and dried in an oven at 105 °C until achieving a constant weight (W_1_). All experiments were conducted in quintuplicate. The MC was calculated using the following equation [20]:

(1) MC (%) = [(W_0_ − W_1_)/W_0_] × 100%

where W_0_ is the initial weight of the film before drying (g) and W_1_ is the film’s constant weight after drying (g).

#### 2.4.3. Light Transmittance of the Film

PP-F and PP-CNC-(35–50%)AEO-F films were cut into rectangular strips measuring 4.0 cm × 1.0 cm, placed on the inner wall of a Shi Ying cuvette, and measured with a UV–visible spectrophotometer (XU-5, Shanghai, China) at the wavelength of 200–700 nm [21]. All experiments were conducted in triplicate.

#### 2.4.4. Film Color Characteristics

The chromaticity of PP-F and PP-CNC-(35–50%) AEO-F film samples was characterized by using a colorimeter (3NH TECHNOLOGY Co., Ltd., SR60, Guangzhou, China), and the parameter values of L (bright–dark), a (red–green), and b (yellow–blue) chromaticity were determined [21]. All experiments were conducted in triplicate.

#### 2.4.5. Thermal Stability of Films

A thermogravimetric analyzer (Mettler Toledo TGA/DSC3+, Greifensee, Switzerland) was used to analyze the thermal stability of PP-F, PP-CNC-AEO-F, and PP-AEO-F films. The samples were weighed to 5–10 mg, the test gas used was nitrogen, the test temperature was set to 30–600 °C, the heating rate was 10.00 K/min, and the flow rate was set to 50 mL/min [20].

#### 2.4.6. Surface Morphology of Films

The surface and cross-sectional structures of PP-F, PP-CNC-(35–50%) AEO-F, and PP-(35–50%) AEO-F were observed by field emission SEM (Apreo 2C, Thermo Fisher Scientific, Waltham, USA) and were treated with gold spraying. The observation was conducted at a voltage of 10 kV and a magnification of 1500 times [22].

#### 2.4.7. FT-IR of Films

FT-IR (Thermo Fisher Nicolet iS20, Waltham, USA) was used to test and analyze different samples. Briefly, Pul and CNC powder were examined by using the potassium bromide tablet method, and the AEO was determined in the transmission mode. The samples of PP-F, PP-CNC-AEO-F, and PP-AEO-F films were measured by attenuated total reflection (ATR) mode [22]. The test conditions were set to the scanning range of 4000–500 cm^−1^, with a cumulative scanning of 64 times.

#### 2.4.8. XRD of Films

The films of Pul, PP-F, PP-CNC-AEO-F, and PP-AEO-F were analyzed by XRD (Rigaku Miniflex, Tokyo, Japan) in the continuous scanning mode, with the scanning range set to 5° to 60° (2θ) and the scanning rate set to 10°/min [11].

### 2.5. Antibacterial and Antioxidant Properties of the Film

#### 2.5.1. Determination of Bacteriostatic Circle and Antibacterial Activity of the Film

The bacteriostatic performance of the film was evaluated by using the bacteriostatic circle method. Briefly, frozen *E.coli* and *S. aureus* strains were inoculated in LB liquid culture medium under aseptic conditions and cultured at 37 °C for 12–24 h to restore the bacterial growth activity, followed by inoculation on a solid plate to obtain a single colony. Then, typical colonies were selected for subsequent amplification. After activation, *S. aureus* and *E. coli* were adjusted to 10^6^ CFU/mL (Maxwell’s turbidimetry). Film disks (15 mm diameter) were sterilized by UV for 30 min, placed on LB agar plates pre-inoculated with 0.1 mL of bacterial suspension, and incubated at 37 °C for 12 h [23]. The inhibition zone diameter was measured with a vernier caliper.

The bacteriostatic rate of the film was determined by using the plate-counting method. The film was cut into a 15 mm disk with a punch, sterilized by UV for 30 min, and placed in LB liquid culture medium inoculated with 10^6^ CFU/mL bacterial suspension, followed by culturing at 37 °C for 12 h under shaking conditions [24]. The co-cultured solution was serially diluted to 10^−6^, and 0.1 mL aliquots were plated on LB agar. Colonies were enumerated after 12 h of incubation at 37 °C. All experiments were conducted in triplicate.

#### 2.5.2. Oxidation Resistance of Films

The DPPH and ABTS radical-scavenging rates of the films were determined by using corresponding kits. The film sample (0.05 g) was extracted with 1 mL of 80% methanol under ultrasonication (200–300 W, 25 °C, 30 min), followed by centrifugation at 12,000 rpm for 10 min at room temperature. The DPPH radical-scavenging rate was determined by measuring absorbance at 517 nm, and the ABTS radical-scavenging rate was determined at 734 nm [12]. All experiments were conducted in triplicate.

### 2.6. Fresh-Keeping Effect of Pickering Emulsion Film of AEO on Mango

Mangoes of the same size, appearance, and maturity were selected. The mangoes were disinfected with 100 mg/L of the NaClO solution for 60 s, washed with distilled water for 60 s, and dried at room temperature. The samples were randomly assigned to the following six groups: control (distilled water), PP-F and PP-CNC-(35–50%) AEO-F. After treatment, the mangoes were stored at 25 ± 1 °C. All experiments were conducted in triplicate.

#### Weight Loss Rate and TSS

The fixed mango samples were regularly weighed to calculate the weight loss rate. Three replicates were set for all assays.

The TSS content of the mango samples was determined regularly by using a digital refractometer, and the pulp juice was taken for determination, with three repetitions [25]. All experiments were conducted in triplicate.

### 2.7. Data Processing and Analysis

Excel 2021 software was used for data statistics and basic processing. IBM SPSS Statistics 27 software was used for data statistical analysis, and one-way analysis of variance (ANOVA) was applied to analyze the significance of the differences between the groups (*p* < 0.05). Different lowercase letters A and B in the chart at the same storage time point indicated a statistically significant difference, and the results were expressed by standard deviation (SD). The images were prepared by Origin 2021.

## 3. Results and Discussion

### 3.1. Droplet Size, PDI, and Zeta Potential of Pickering Emulsions of Different Concentrations of AEO

**Figure 1:**
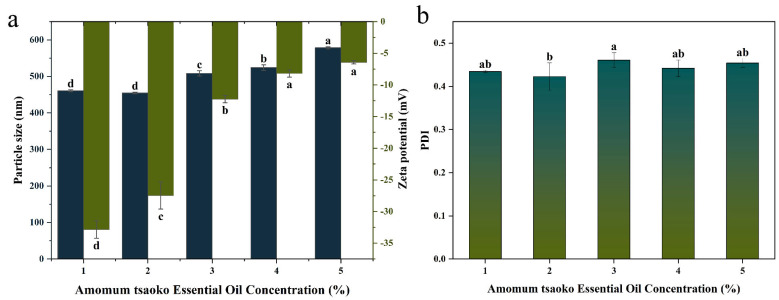
Droplet size and zeta potential (**a**) of essential oil of *Amomum tsaoko* Pickering emulsion at different concentrations; PDI of Pickering emulsion of *A. tsaoko* essential oil at different concentrations (**b**). ^a–d^ refers to the significant differences between different groups (*p* < 0.05).

The concentration of AEO plays a key role in the formation and stability of Pickering emulsion. Different AEO concentrations (1–5%) were evaluated to determine the optimal formulation of Pickering emulsions. The average particle size, zeta potential, and PDI of these emulsions containing different AEO concentrations are presented in Figure 1. The average droplet size of the emulsion was the smallest at 1% and 2% AEO concentration, whereas the PDI was the lowest at the 1% concentration, indicating reduced particle aggregation and a more uniform distribution of droplets [26]. Under conditions of a low oil phase and a high CNC ratio, the surface coverage of oil droplets was more complete, which promoted the formation of a continuous permeable fiber network in the aqueous phase and inhibited agglomeration [27,28]. In addition, the absolute value of the zeta potential of the emulsion decreased from 32.83 to 6.44 as the EO concentration increased, indicating reduced stability of the emulsion system with an increase in the AEO concentration. A higher absolute value of the zeta potential indicates stronger electrostatic repulsion, which prevents droplet aggregation and enhances stability. At AEO concentrations of 1% and 2%, the emulsion’s zeta potential values were lower than −25 mV. Emulsions with zeta potential values lower than −25 mV are generally considered sufficiently stable [29], which prevents particle sedimentation and ensures excellent electrostatic stability.

### 3.2. Study on Microstructure and the Stability of AEO Pickering Emulsion

**Figure 2:**
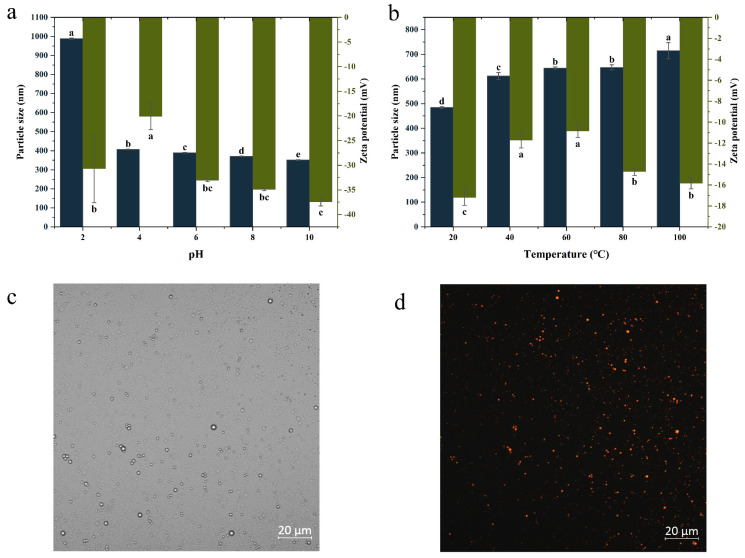
pH stability of *Amomum tsaoko* essential oil Pickering emulsion (**a**); temperature stability (**b**); microscopic morphology (scale is 20 μm) (**c**,**d**). ^a–e^ refers to the significant differences between different groups (*p* < 0.05).

The 2% AEO Pickering emulsion (AEO–CNC), identified as optimal in preliminary experiments, was used to determine its pH stability and thermal stability. Changes in pH significantly affect the charge distribution and hydrophilic–hydrophobic balance of stabilizing particles, thus changing their adsorption behavior at the oil–water interface and influencing the droplet size and stability of the emulsion system. The stability results are presented in Figure 2a. The overall particle size remained between 350 nm and 450 nm at pH 4–10. As the pH increased above 4, the particle size gradually decreased, while the absolute zeta potential value gradually increased, reflecting the improved stability of the emulsion. This improvement may be attributed to enhanced electrostatic repulsion between negatively charged nano-cellulose particles. The deprotonation of CNC functional groups at higher pH increases surface charge, promotes the interfacial adsorption of CNC, inhibits droplet coalescence, and enhances overall stability [28]. At pH 2, the particle size was the largest (988.13 nm), and the emulsion was agglomerated with droplets under strong acidic conditions. The AEO–CNC emulsion exhibited good stability within the pH range of 4–10. As shown in Figure 2b, after heat treatment at 20 °C, 40 °C, 60 °C, 80 °C, and 100 °C, the emulsion particle size ranged from 450 nm to 750 nm. As the temperature increased, the emulsion particle size increased to a certain extent. The absolute zeta potential value was the highest after heat treatment at 20 °C. The results show that AEO–CNC exhibits optimal thermal stability at 20 °C after heat treatment for 30 min. In this study, the microstructure of the AEO–CNC emulsion was investigated using confocal laser scanning microscopy. The observation results in Figure 2c indicate that the emulsion droplets are spherical in morphology [30]. As shown in Figure 2d, droplets of varying sizes exhibiting red fluorescence signals were identified as oil droplets. Overall, these experimental findings are in good agreement with the results reported by Zhao and Akgönüllü et al. [18,31].

### 3.3. Mechanical Properties, Moisture Content, and Light Transmittance of the Film

**Figure 3:**
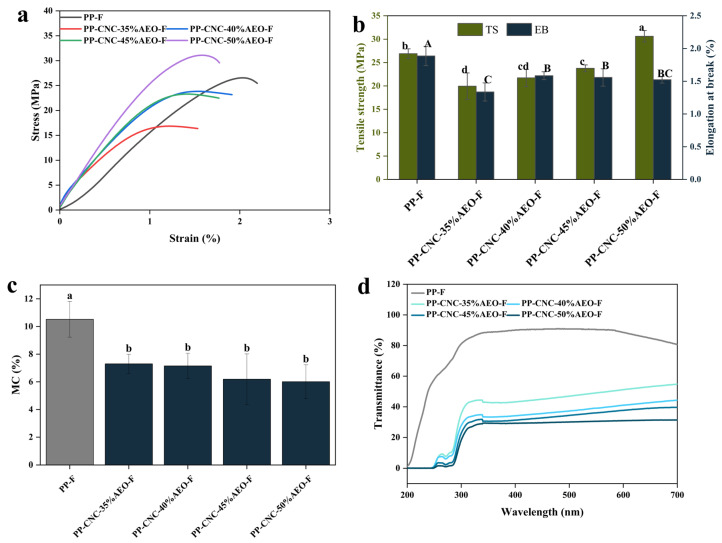
Stress–strain curves, tensile strength, and elongation at break (**a**,**b**) of PP-F and PP-CNC-(35–50%) AEO-F; moisture content (**c**); light transmittance (**d**). ^a–d^ ^and A–C^ refers to the significant differences between different groups (*p* < 0.05).

Mechanical properties are key indicators for evaluating films used in food packaging and are closely related to their structural characteristics. Figure 3a,b show the stress–strain curves, tensile strength, and elongation at break of films, including PP-F and PP-CNC-(35–50%) films. For the PP film, the TS and EAB values were 26.90 ± 1.04 MPa and 1.89% ± 0.15%, respectively. As the AEO–CNC content increased from 0% to 45%, the TS of composite films decreased from 26.90 ± 1.04 MPa to 23.76 ± 0.73 MPa. This reduction can be attributed to the disruptive effect of AEO droplets on the polymer matrix. At these lower concentrations, AEO interferes with intermolecular interactions within the Pul chains, weakening the hydrogen bonding and reducing cohesion; at the same time, the reinforcing effect of CNC is inadequate to compensate for this disruption [32]. Supplementing the Pickering emulsion of clove EO was found to slightly reduce the TS of carrageenan/agar-based films [33]. A similar reduction in TS was reported with the addition of citrus EO-loaded Pickering emulsion to pectin films [34]. However, at an AEO–CNC content of 50%, TS increased to 30.62 ± 1.23 MPa, which was significantly higher than the TS of the PP film. The increase in TS can be attributed to the hydrogen-bonding interactions between AEO–CNC and the PP matrix, which counteract the damage caused by the incorporation of AEO into the film. Similar findings were reported by [35], where the incorporation of Pickering emulsions of CEO into the film increased its TS value due to the improved interfacial interaction between the polymer matrix and PE that counteracted the destructive effects of CEO on the continuous structure of the film [36]. As the emulsion content increased, the CNC content in the film increased, gradually leading to improved mechanical properties. By contrast, the EAB gradually decreased with an increase in AEO–CNC content. Excessive incorporation of emulsion reduces the uniformity of the film and leads to aggregation of emulsion droplets, ultimately decreasing the internal continuity of the film [37]. These findings are consistent with the SEM analysis results. The ratio and content of AEO and CNC are critical for optimizing the structure and interaction of film components.

In Figure 3c, the inherent water content of the polysaccharide results in the highest moisture content of the PP film (10.5%). As the AEO–CNC content increased from 35% to 50%, the moisture content of the film tended to decrease. The moisture content was the lowest (6.01%) at an AEO–CNC content of 50%. This decrease is attributed to the hydrophobic nature of EOs, which reduces the availability of hydrophilic groups in the film and limits water absorption and solubility [38]. Moreover, intermolecular interactions between the polymer matrix and nanoemulsion further reduce water absorption [33]. Additionally, the density of the film increases with an increase in dry matter content, thereby increasing film hydrophobicity and decreasing water content [35].

The light barrier properties of PP-F and PP-CNC-(35–50%) AEO-F films were studied using visible light transmittance measurements. Lower transmittance indicates better light-blocking ability, thus reducing photooxidation-induced food deterioration. The UV–visible transmission curve presented in Figure 3d shows that the UV transmittance of PP-CNC-(35–50%) AEO-F films was below 30% in the 200–400 nm range, while the PP film exhibited the highest transmittance (88.51%) at 600 nm. The transmittance of PP-CNC-(35–50%) AEO-F composite films was low at 600 nm. This UV-blocking effect is attributed to aromatic compounds and phenolic hydroxyl groups in AEO, which absorb and scatter UV radiation [39]. Moreover, the light scattering of Pickering emulsion droplets is enhanced because of the differences in refractive index [40]. The higher the emulsion concentration, the higher the density and granule of the film, which further affects the molecular structure and reduces light transmittance [41]. Compared with PP films, the lower UV transmittance and higher opacity of PP-CNC-(35–50%) AEO-F films reduce lipid oxidation and light-induced degradation of packaged food, making them an ideal choice for protecting photosensitive foods.

### 3.4. Film Chromaticity

The color properties of packaging films are important in the food industry as they directly affect consumer perception and acceptance. The color parameters L, a*, and b* are shown in Table 2. The PP film exhibited the lowest L* value, whereas the PP-CNC-50%AEO-F film exhibited the highest L* value, and a significant difference in brightness was observed between them (*p* < 0.05). Incorporating AEO–CNC significantly increased the brightness of the composite film and reduced its coloration intensity. The a* and b* values of PP-CNC-(35–50%) AEO-F films gradually decreased, indicating a shift toward blue-green tones.

### 3.5. Thermogravimetric, FT-IR, and XRD of Films

**Figure 4:**
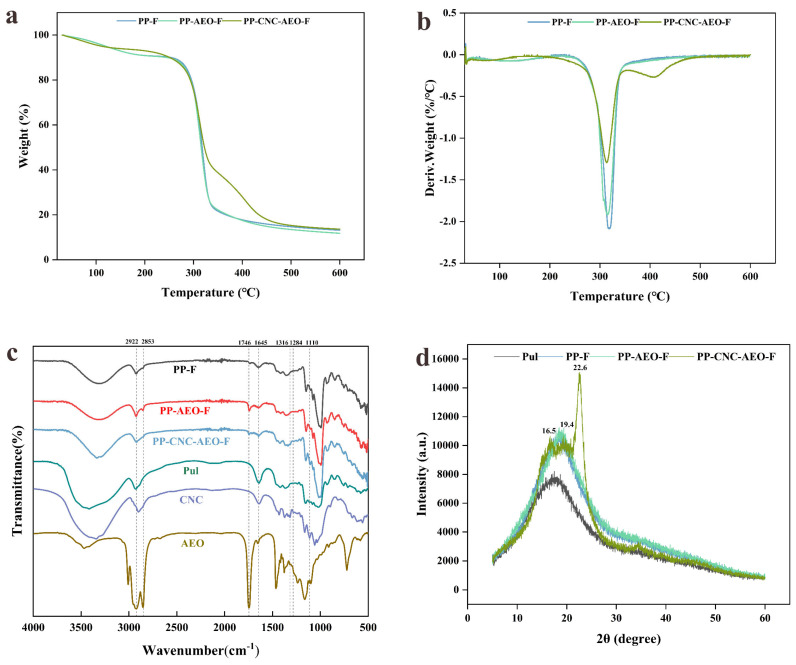
Thermogravimetric, Fourier infrared and X- ray diffraction of films (**a**–**d**).

Thermal stability is a key indicator of the performance of packaging materials, as it directly affects the structural integrity and performance stability of films during processing and storage. Figure 4a,b show the TG and DTG curves of each film, respectively. All films exhibited initial weight loss in the range of 30–90 °C, which was attributed to the breaking of intermolecular/intramolecular hydrogen bonds, dehydration, and the volatilization of compounds from EO. Weight loss in the second stage (90–350 °C) was mainly associated with the thermal degradation of glycerol, volatilization of bound water, and the PP matrix. Notably, only the PP-CNC-AEO-F film exhibited a third stage of weight loss at 350–480 °C, attributed to CNC thermal decomposition [42]. At 30–450 °C, the overall weight loss rate of PP-CNC-AEO-F films was significantly lower than that of the other two films. Although the maximum degradation temperatures of the three films were mostly the same, the maximum degradation rate of PP-CNC-AEO-F films was the lowest. Compared with PP-F and PP-AEO-F films, PP-CNC-AEO-F composite films exhibited higher thermal stability, which may be attributed to the excellent thermal stability of cellulose. Furthermore, the residual mass of PP-AEO-F films was lower than that of PP-F films, likely due to the decrease in matrix content after EO incorporation [39]. Studies have also reported that thermal degradation of polymer systems with high crystallinity is usually more difficult [43]. In general, incorporation of Pickering emulsions of AEO effectively improves the thermal stability of films.

Figure 4c presents the FT-IR spectra of Pul, CNC, AEO, PP-F, PP-AEO-F, and PP-CNC-AEO-F. The absorption peaks of Pul powder at 3395 and 2922 cm^−1^ correspond to the stretching vibrations of O-H and C-H, respectively, while the absorption peak at 1645 cm^−1^ corresponds to the stretching vibration of conjugated carbonyl (C=O). All composite films exhibited characteristic absorption bands similar to those of Pul, with a broad band at 3471–3199 cm^−1^ corresponding to O-H stretching vibrations. The absorption peaks of all films shifted toward low wavenumbers (red shift), indicating that the films form intermolecular hydrogen bonds between hydroxyl groups of materials [44]. For PP-AEO-F and PP-CNC-AEO-F films, the characteristic absorption peaks of AEO were detected. The peak at 2853 cm^−1^ corresponds to aliphatic and unsaturated hydrocarbons (terpenoids) in AEO [45,46], while the peak at 1746 cm^−1^ corresponds to the stretching vibration of aromatic aldehyde carbonyl groups [42]. In PP-CNC-AEO-F films, these peaks appeared weaker, possibly because AEO molecules were embedded within the polysaccharide matrix and involved in hydrogen bonding, which restricted their molecular vibrations [47]. In addition, with increasing emulsion content, absorption peaks at 1316, 1284, and 1110 cm^−1^ gradually appeared, corresponding to the characteristic vibrations of CNC. The absorption peak at 1316 cm^−1^ is associated with the vibration of hydroxyl (–OH) bending in cellulose [48], the tensile absorption peak at 1284 cm^−1^ corresponds to the vibration of the lignin carboxylate bond [47], and the spectral absorption peak at 1110 cm^−1^ corresponds to the stretching vibration of the important chemical bond C–O–C between glucose units in cellulose [49]. This observation further confirmed that the emulsion was successfully incorporated and dispersed within the polysaccharide matrix, and the peaks in the film could all correspond to CNC, Pul, and AEO, indicating that no chemical reaction occurred as no new substances were produced after mixing.

Crystallinity is closely related to the molecular arrangement and characteristics of composite films. As shown in Figure 4d, Pul, PP-F, and PP-AEO-F films showed broad diffraction peaks at 2θ = 17.6° and 18.4°, respectively, indicating that Pul and the films are mainly amorphous structures. With the increase in CNC content, additional diffraction peaks appeared in PP-CNC-AEO-F films at 2θ = 16.5°, 19.4°, and 22.6°, which corresponded to the crystal planes 110, 101, and 200 of the films [50]. Studies have reported that EO destroys the crystal structure of films [51,52]. However, CNC counteracts this effect and enhances structural order in the film through its own and stronger force [53]. Nanoparticles help to stabilize and promote the ordered structure [38], and the improvement in the crystallinity of the film enhances the physical properties of the composite film [53]. However, the noise in all XRD spectra proves that the crystallinity of the film matrix is poor [46].

### 3.6. SEM of Films

**Figure 5:**
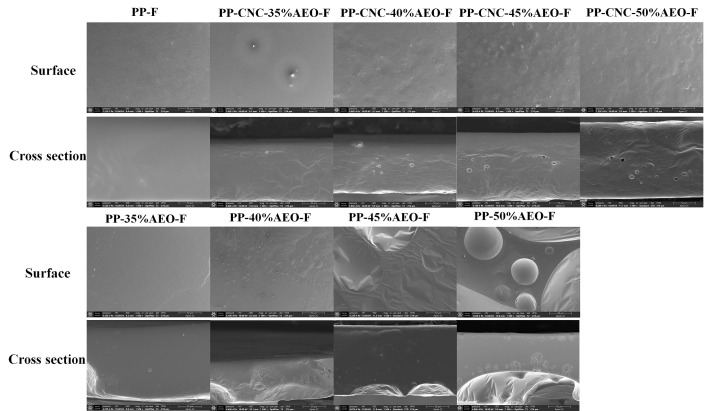
Scanning electron microscope images of film surface and cross-section.

Figure 5 shows the surface and cross-sectional morphologies of PP-F, PP-CNC-(35–50%) AEO-F, and PP-(35–50%) AEO-F films. The PP-F film exhibited a smooth, flat, and continuous structure. With increasing AEO content, bubbles appeared on both the surface and cross-section of PP-(35–50%) AEO-F films, and their number gradually increased. Some bubbles were shrinking, likely due to the migration of EO droplets of AEO to the film surface and solvent evaporation during film formation. Excessive air pockets may undergo structural collapse upon rupture [54], disrupting intermolecular interactions between polysaccharides in the matrix. By contrast, as the AEO–CNC content increased, the surface of PP-CNC-(35–50%) AEO-F films became denser and more uniform, with no obvious cracks. However, when the number of particles increased, the surface became rough. Fine pores were observed in the cross-section, indicating the successful incorporation of the Pickering emulsions of AEO into the composite film system [16].

### 3.7. Bacteriostasis and Oxidation Resistance of Films

**Figure 6:**
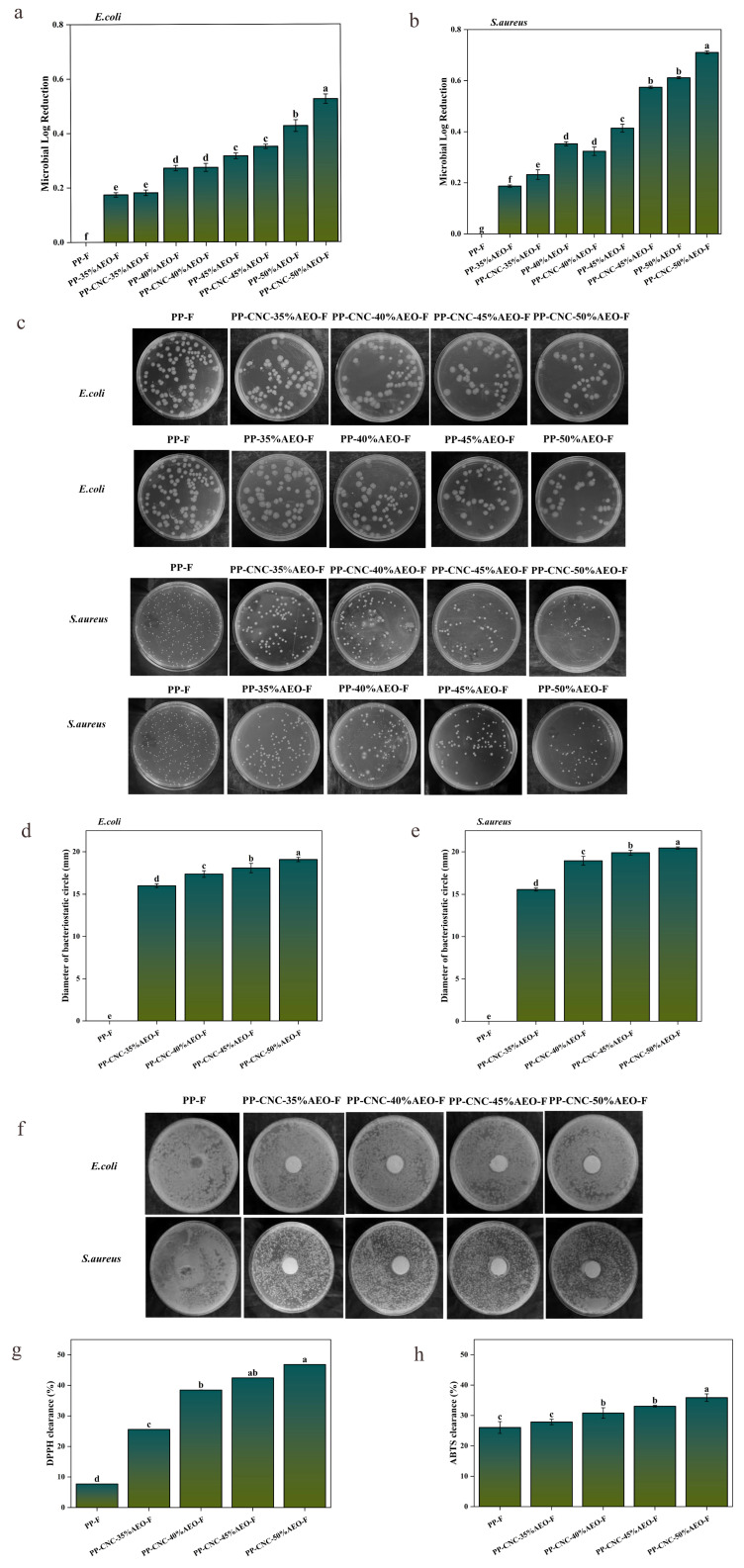
Antibacterial activity of film against *E. coli* and *S. aureus*; (**a**–**c**) bacteriostatic circle diagram (**d**–**f**) of the film against *E. coli* and *S. aureus*; oxidation resistance diagram of thin film (**g**,**h**). ^a–g^ Refers to the significant differences between different groups (*p *< 0.05).

The antibacterial activities of PP-F, PP-CNC-(35–50%) AEO-F, and PP-(35–50%) AEO-F films against *E. coli* and *S. aureus* were evaluated by using the plate-counting method. As shown in Figure 6a–c, for *E. coli*, PP-CNC-(35–50%) AEO-F films displayed logarithmic cycle reduction ranging from 0.181 to 0.525, whereas PP-(35–50%) AEO-F films exhibited logarithmic cycle reduction of 0.174–0.426. For *S. aureus*, PP-CNC-(35–50%) AEO-F films showed logarithmic cycle reduction of 0.232–0.710, and PP-(35–50%) AEO-F films ranged from 0.187 to 0.611. The PP-F control exhibited negligible antibacterial activity against both bacteria. Although the observed logarithmic cycle reduction indicated a moderate inhibitory effect, the films prepared using Pickering emulsions of AEO generally demonstrated slightly higher antibacterial activity than those prepared with AEO alone. This difference may be observed due to the instability and volatility of the EO, which increases the short-term release of EO within the emulsion system. The matrix of the emulsion composite film serves as an effective barrier for the EO, thereby reducing its instability and allowing the continuous release of antibacterial substances to ensure a lasting antibacterial effect [10]. Among them, PP-CNC-50%AEO-F films exhibited the most significant antibacterial activity.

Moreover, the antibacterial performance of PP-F and PP-CNC-(35–50%) AEO-F films was further verified using the inhibition zone method. As shown in Figure 6d–f, the inhibition zone diameters of PP-F and PP-CNC-(35–50%) AEO-F films against *E. coli* were 0, 16.01, 17.36, 18.08, and 19.08 mm, respectively, while those against *S. aureus* were 0, 15.57, 18.94, 19.89, and 20.45 mm, respectively. These results indicate that the diameter of the inhibition zone increased with an increase in the AEO content of Pickering emulsions, indicating enhanced antibacterial ability. In addition, PP-(35–50%) AEO-F films melted during the experiment, indicating poorer structural stability. The antibacterial activity of the films is mainly attributed to AEO, which can disrupt the structure of biofilms and damage the integrity of cell walls and membranes [55], leading to the leakage of intracellular electrolytes and macromolecules [4]. In addition, AEO can reduce the activities of bacterial ATPase and G6PDH and cellular ATP content. It also inhibits the pentose phosphate pathway, thus interfering with the energy metabolism and respiratory metabolism of bacteria and ultimately leading to bacterial death [5]. The research also reported that AEO exerts a stronger inhibitory effect on Gram-positive bacteria than Gram-negative bacteria, mainly because the cell wall of Gram-positive bacteria is primarily composed of peptidoglycan, which allows hydrophobic molecules to penetrate the cell wall and cytoplasm and cause bacterial death [10].

Developing packaging materials with antioxidant activity is an effective strategy for regulating fruit and vegetable ripening and delaying aging. The present study analyzed the antioxidant properties of PP-F and PP-CNC-(35–50%) AEO-F films. In Figure 6g,h, as the AEO–CNC concentration increased, the DPPH radical-scavenging rate of the five groups of samples exhibited an overall upward trend. The scavenging rate of PP-F films was always low (approximately 7.72%), while all PP-CNC-(35–50%) AEO-F films exhibited strong antioxidant activity, especially the PP-CNC-50%AEO-F films, which had the highest DPPH-free radical-scavenging rate (reaching 46.83%). This improvement is attributed to the effective encapsulation of AEO within the CNC-stabilized emulsion and its synergistic interaction with the intrinsic antioxidant activity of Pul [56]. Consistent with the DPPH scavenging results, PP-F films also exhibited moderate ABTS radical-scavenging activity (26.04%), which confirmed the antioxidant activity of Pul [56]. As the emulsion concentration increased, the ABTS radical-scavenging activity of the films gradually increased, thus significantly enhancing the overall antioxidant activity of the coating. This performance improvement is mainly attributed to the presence of rich bioactive components in AEO, such as phenols, flavonoids, and terpenoids [57], which efficiently scavenge free radicals through electron transfer and hydrogen atom donor mechanisms.

### 3.8. Fresh-Keeping Effect of AEO Pickering Emulsion Films on Mango

**Figure 7:**
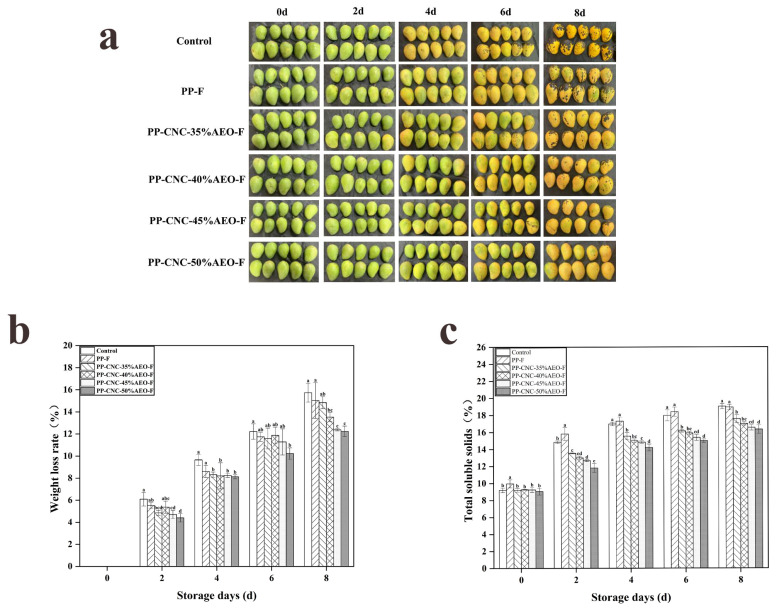
Effects of film treatment on appearance (**a**), weight loss rate (**b**), and soluble solid content (**c**) of mango. ^a–e^ refers to the significant differences between different groups (*p* < 0.05).

In Figure 7a, during storage, all mango samples gradually ripened during storage; however, the degree of ripening and decay in samples coated with AEO-CNC films was significantly lower than that in the control and PP-F groups. The coating group added with AEO-CNC exhibited a high inhibitory effect on the formation of black spots on the mango surface because of its high antibacterial activity [26].

As shown in Figure 7c, the TSS of mango increased continuously during storage, with the initial content being approximately 9.5%. During the whole storage process, the TSS content of the control and PP-F groups was significantly higher than that of other coating treatment groups. As the AEO–CNC content increased, the inhibitory effect of the coating on TSS content gradually increased, indicating that the coating treatment effectively delayed the accumulation of soluble solids in mango and slowed postharvest ripening [2]. TSS mainly included sugars, organic acids, and minerals. The increase in TSS content was related to the hydrolysis of pectin [58] and other substances during storage and water loss. The coating treatment inhibited the metabolic activity of mango, reduced water evaporation, and decreased the weight loss rate (Figure 7b) [2], thus delaying the increase in TSS content.

## 4. Conclusions

In this study, an active film (PP-CNC-AEO) based on Pul and incorporating Pickering emulsions of AEO was prepared. The average particle size of AEO-loaded Pickering emulsions was 454.73 ± 2.45 nm. Compared with the pure Pul film, the composite film exhibited higher mechanical strength, UV-blocking performance, and thermal stability. The CNC-stabilized emulsion effectively encapsulated AEO, thus providing the film with unique antioxidant and antibacterial properties. The microstructure of the film revealed that AEO-loaded Pickering emulsions were uniformly dispersed in the film, promoting the stable formation of a dense film through strong entanglement and hydrogen bonding. Incorporation of AEO–CNC improved film crystallinity. The prepared film prolonged the shelf life of mangoes, achieving a 4-day extension relative to the control group. This enhanced preservation performance underscores the film’s ability to effectively retard postharvest deterioration and improve the storage stability of mangoes. Thus, the prepared film showed great potential in fruit and vegetable preservation, and the study findings serve as a reference for the development and application of polysaccharide-based films for food packaging. However, there are several limitations to the present study, based on which it is recommended that future studies should therefore focus on comprehensive evaluation of the emulsion storage stability, quantitative determination of AEO retention during film formation, and detailed investigation of AEO-release kinetics using appropriate release models. In addition, the performance of these films warrants further validation under real food-packaging conditions, using different food systems, storage environments, shelf-life extension, and microbial inhibition that are encountered during real-time practical applications. Such investigations will provide a deeper understanding of the structure–function relationship of the films and thereby further support their potential as active food-packaging materials.

## Figures and Tables

**Table 2:** Thin film chromaticity table. ^a–d^ refers to the significant differences between different groups (*p* < 0.05).

	L	a*	b*
PP-F	41.57 ± 1.97 ^d^	0.70 ± 0.23 ^a^	1.16 ± 0.19 ^a^
PP-CNC-35%AEO-F	44.56 ± 1.22 ^c^	−0.30 ± 0.14 ^b^	0.66 ± 0.48 ^ab^
PP-CNC-40%AEO-F	46.57 ± 0.89 ^bc^	−0.57 ± 0.14 ^bc^	0.49 ± 0.17 ^bc^
PP-CNC-45%AEO-F	48.65 ± 1.20 ^ab^	−0.59 ± 0.20 ^bc^	0.06 ± 0.08 ^cd^
PP-CNC-50%AEO-F	50.43 ± 2.23 ^a^	−0.65 ± 0.13 ^c^	−0.30 ± 0.38 ^d^

## Data Availability

The original contributions presented in this study are included in the article. Further inquiries can be directed to the corresponding author.
